# Epi-Drugs in Heart Failure

**DOI:** 10.3389/fcvm.2022.923014

**Published:** 2022-07-13

**Authors:** Era Gorica, Shafeeq A. Mohammed, Samuele Ambrosini, Vincenzo Calderone, Sarah Costantino, Francesco Paneni

**Affiliations:** ^1^Center for Molecular Cardiology, University of Zürich, Schlieren, Switzerland; ^2^Department of Pharmacy, University of Pisa, Pisa, Italy; ^3^Department of Cardiology, University Heart Center, Zurich, Switzerland; ^4^Department of Research and Education, University Hospital Zurich, Zurich, Switzerland

**Keywords:** epigenetics, cardiovascular diseases, epi-drugs, heart failure, non-coding RNAs

## Abstract

Unveiling the secrets of genome’s flexibility does not only foster new research in the field, but also gives rise to the exploration and development of novel epigenetic-based therapies as an approach to alleviate disease phenotypes. A better understanding of chromatin biology (DNA/histone complexes) and non-coding RNAs (ncRNAs) has enabled the development of epigenetic drugs able to modulate transcriptional programs implicated in cardiovascular diseases. This particularly applies to heart failure, where epigenetic networks have shown to underpin several pathological features, such as left ventricular hypertrophy, fibrosis, cardiomyocyte apoptosis and microvascular dysfunction. Targeting epigenetic signals might represent a promising approach, especially in patients with heart failure with preserved ejection fraction (HFpEF), where prognosis remains poor and breakthrough therapies have yet to be approved. In this setting, epigenetics can be employed for the development of customized therapeutic approaches thus paving the way for personalized medicine. Even though the beneficial effects of epi-drugs are gaining attention, the number of epigenetic compounds used in the clinical practice remains low suggesting that more selective epi-drugs are needed. From DNA-methylation changes to non-coding RNAs, we can establish brand-new regulations for drug targets with the aim of restoring healthy epigenomes and transcriptional programs in the failing heart. In the present review, we bring the timeline of epi-drug discovery and development, thus highlighting the emerging role of epigenetic therapies in heart failure.

## Introduction

The new trend is epigenetics, a field advancing with more galloping steps than technology. A heightened interest in epigenetics is followed by advanced insights into health and diseases, especially in those affected by heritable changes in gene expression patterns that are not accompanied by alterations of the DNA sequence (1, 2). The chemical modifications occurring to the genome to regulate gene expression are referred to as epigenetic changes. Methylation of CpG islands at the level of DNA is the most studied epigenetic modification. Methylation occurs within gene promoters and consists of an addition of methyl groups to the DNA molecule which represses gene transcription when it is located in close proximity to a gene (e.g., gene promoter or gene body) (3). Other epigenetic modifications are also important and worth focusing the studies on. Histone modifications and non-coding RNAs (ncRNAs) are as critical as they play a crucial role in disease development by controlling gene expression (4). Methylation, acetylation, ubiquitination, phosphorylation, and ADP-ribosylation are considered post-translational modifications (PTM) that occur on histone levels thus influencing the histone conformation and, hence, chromatin accessibility (5). Whereas ncRNAs are found to participate in many physiological and pathological processes by modulating the transcriptional output from the genome. Together with DNA methylation and histone PTM, they are recognized as powerful regulators of gene expression and are heavily implicated in human diseases (6–8).

Importantly, compelling evidence has shown that epigenetic signals promote phenotypic changes by modulating genes controlling cardiovascular homeostasis. Once acquired, epigenetic changes can be relatively stable and persist over time. However, these changes have shown to be reversible upon cessation of different stimuli, and most importantly, they are amenable to pharmacological intervention. The possibility to pharmacologically erase detrimental epigenetic changes to prevent disease is fascinating and is gaining increasing attention. The epi-drug era has begun and with it the competition among research groups and (big)pharmas for the development of novel and effective drugs. New approaches for the development of new therapeutic strategies aim at targeting various epigenetic factors that introduce, recognize, or remove modifications at the level of DNA/histones. Three families of epigenetic proteins – readers, writers, and erasers – are on focus as druggable targets; they can be conveniently manipulated thus highlighting the importance of developing these drugs. Successful development and handling of epi-drugs will help to define better policies for the treatment of a plethora of diseases (9, 10).

## From Pharmacogenetics and Pharmacogenomics to Epi-Drugs in Cardiovascular Diseases

Pythagoras became the pioneer of pharmacogenetics bringing the very first evidence when he observed that fava beans resulted in potentially fatal hemolysis in some, but not all individuals. Nowadays this phenomenon is recognized as the most trivial enzymatic deficiency and is linked to glucose-6-phosphate dehydrogenase deficiency (G6PD) (11). The term “pharmacogenetics” wasn’t used until the 1950s when Vogel coined the term in reference to the discovery of some enzyme polymorphisms and dedicated a whole section in his chapter on “modern problematics of human genetics” (12). Albeit initially conceived as an esoteric matter, numerous studies have fashioned this field of research throughout the years. Pharmacogenetics can come in help to solve the conundrums as to why different people, but also different populations, respond variably to the same medication. Studies have revealed that gene mutation is not the reason for these responses, but the alteration in gene-editing enzymes is (13, 14). The suffix “omics,” which is often used interchangeably with the former one, is added to the term. Both fields are important as they focus on the study of variability in drug response due to heredity and are seen as promising in the improvement of drug therapy. Progressive advances in pharmacogenetics gave rise to pharmacogenomics (15, 16). It is known that epigenomics influences drug development and approval. Although the relevance and the contribution of these fields to personalized medicine remain to be studied, there is ample evidence to support its efficacy and effectiveness. The FDA itself strongly supports the progress in these fields (17). The variability in response to different medical treatments or drugs, regardless of different factors (environmental, biological, or genetic), requires further studies which can easily take advantage of the established background in pharmacogenetics and pharmacogenomics (16, 17). In large-scale, epigenomic studies are of growing importance. The epigenetic drug discovery and drug development processes are moving the attention toward epi-drugs as a major factor with potential use in precision medicine (18). On the other hand, understanding the genetics’ contribution to drug failure and drug toxicity may help not only in the development of new treatments but also to improve the safety, efficacy, and costs of already existing drugs. Yet, integrating pharmacogenetics into clinics remains a challenging motion (18). A crucial step is the establishment of pharmacogenetic networks that can help in the collection and centralization of pharmacogenetic information. More than ever, we need pharmacogenomic inspections to study gene patterns and disease-causing genes. These investigations aspire to look closely at the expression of the whole sets of genes to further consider modifications through drug manipulation (14, 18).

A major public health problem, and the leading cause of mortality worldwide is cardiovascular disease (CVDs) with heart failure (HF) dominating the scene (19). HF currently affects 26 million people worldwide and 15 million people only in Europe. Most importantly, a 46% in HF prevalence is expected by the year 2030. This growing burden is certainly the result of a poor understanding of the disease and the factors involved in its development. Several studies have shown that non-cardiomyocyte cell populations significantly contribute to cardiac remodeling in HF. Recent studies now point to the activation of resident fibroblasts as the underlying cause of fibrosis. However, *de novo* generation of fibroblasts from endothelium and circulating hematopoietic cells has also been proposed to significantly contribute to fibrosis in the setting of HFpEF. Through single-cell RNA-seq, spatial transcriptomics, and genetic perturbation, a recent study found that high-temperature requirement, a serine peptidase 3 (Htra3) is a critical regulator of cardiac fibrosis and HF by maintaining the identity of quiescent cardiac fibroblasts through degrading transforming growth factor-β (TGF-β) (20). Together with fibroblasts, factors secreted by (dysfunctional) endothelial cells have shown to modulate cardiomyocytes hypertrophy, contractility and fibrosis, thus accelerating the progression toward HF (21). Future studies should unveil the role of epigenetic signals in the functional crosstalk among different cell types in the pathogenesis of HF.

Actually, epigenetic changes have an impact on modulating chromatin accessibility to transcription factors and gene expression. Modulation of epigenetic signals and manipulation of alterations in chromatin modifying enzymes may represent a new effective therapy for HF patients (22). In this perspective, it is crucial to head the treatments toward medicine based on one’s genetic composition, disease phenotype, and molecular makeup. A useful tool to identify patients who can benefit from specific drugs is genome mapping (23). Epigenetic alterations are proven to be restored and epigenetic therapies have arisen. Suitable epi-drugs candidates seem to be of potential use in CVDs in general and HF in particular (24, 25). Brand-new epi-drugs have started to gain importance. They are either in process of preclinical studies or being tested in clinical trials. Moreover, various existing cardiovascular drugs were recently shown to have possible epigenetic effects. The use of epi-drugs in the clinical arena might enable personalized therapies and could help implement existing cardiovascular therapies to improve HF care and patient prognosis (26).

## Epigenetic Networks in Heart Failure

**FIGURE 1:**
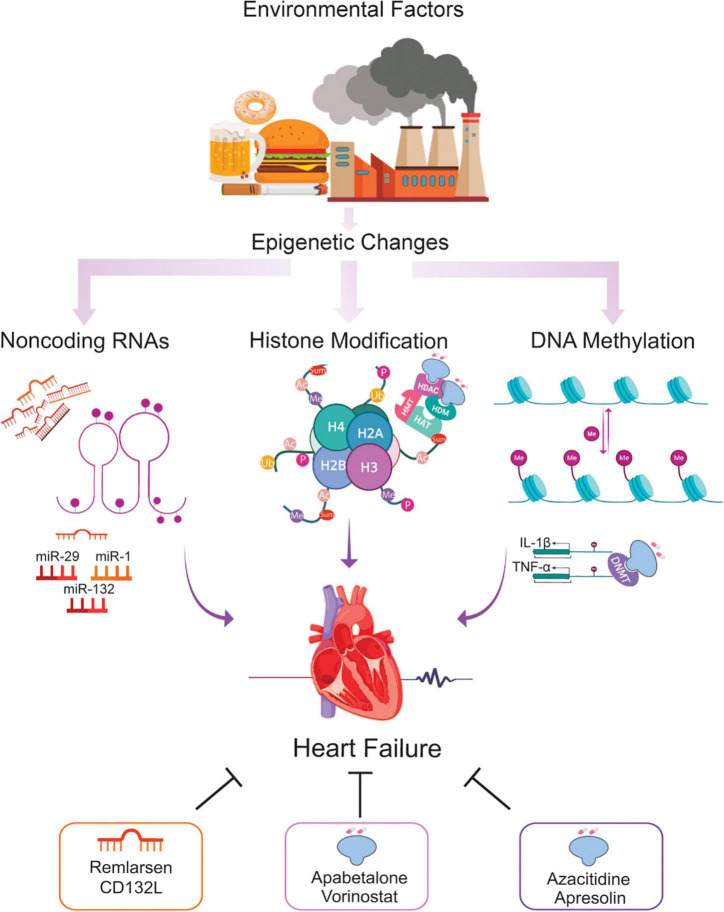
Epigenetic changes potentially involved in the HF and the role of epi-drugs. Throughout life, numerous environmental factors induce epigenetic signals which alter the expression of genes implicated in the development of HF. Alterations in DNA methylation, histone modifications, and ncRNA elicit transcriptional changes leading to cardiac remodeling, fibrosis, and microvascular dysfunction, key hallmarks of the failing heart. Using epi-drugs to target chromatin-modifying enzymes, or employing short oligonucleotides to mimic or antagonize relevant ncRNA appear to be a promising strategy for a personalized management of patients at risk of developing HF. *Ac = acetylation, Ub = ubiquitination, Sum = sumoylation, Me = methylation, P = phosphorylation, HAT = histone acetyltransferase, HMT = histone methyltransferase, HDAC = histone deacetylase, HDM = histone demethylase, DNMT = DNA methyltransferase.

**FIGURE 2:**
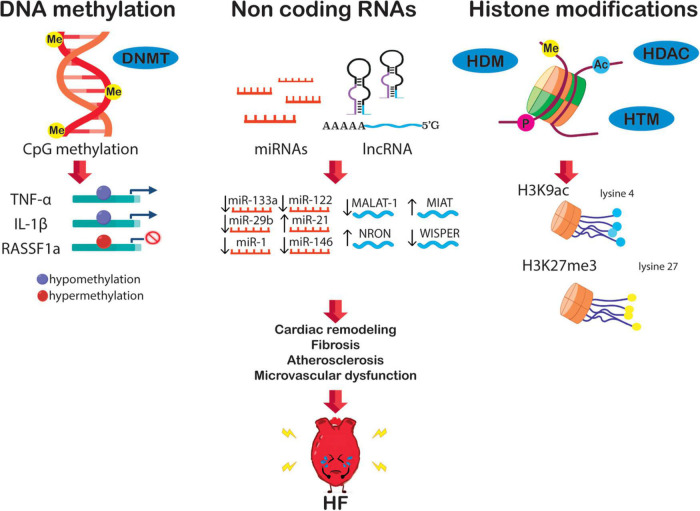
The epigenetic network in HF is potentially orchestrated by alterations of DNA methylation, non-coding RNA, and histone modification. These alterations boost transcriptional changes leading to crucial HF features, i.e., diabetes, fibrosis, cardiac remodeling, atherosclerosis, and microvascular dysfunction.

Epigenetics is essential for normal organismal development and cellular functioning. Exposure to environmental stressors and an unhealthy lifestyle can alter epigenetic modifying enzymes and thus the chromatin landscape (Figure 1) (27). Three major risk factors for the development of HF – diabetes, obesity, and aging – are seen to increase the prevalence and mortality of the disease. Noteworthy, these conditions are linked to epigenetic changes (28–31). These changes can cause detrimental modifications of the epigenetic landscape, consequently increasing the risk of, or directly provoking, HF (Figure 2). Alterations of gene expression play a dominant role in governing cardiac remodeling and disease pathogenesis. Found in such conditions, the heart itself undergoes functional and structural remodeling that encompasses considerable transcriptional reprogramming (32). By this background, understanding the mechanisms governing gene expression is of paramount importance. Many transcription factors don’t change in their abundance but only in their activity, suggesting a pivotal role of posttranslational events (33, 34). Consequently, both, direct modifications of transcription factors and modifications of chromatin to alter gene accessibility play a role in affecting enzyme activity and transcriptional reprogramming. This is explained by the broad importance gained by the regulation of transcript stability and transcript translation through microRNAs. DNA/histone modifications and non-coding RNA remain molecular transducers of environmental stimuli to control gene expression. Evidence in the field indicates that epigenetic regulation could be an important biological layer that actively participates in CVD and HF phenotypes, and its modulation could be a promising and innovative therapeutic tool to improve the diagnosis, prognosis, and treatment of HF (35).

### DNA/Histone Modifications

DNA can be easily modified *via* the addition of a methyl group to cytosine residues. This chemical reaction is catalyzed by DNA methyltransferases (DNMTs). Unmethylated cytosines are methylated by DNMT3B and DNMT3A known as “*de novo* DNMTs,” whereas DNMT1 is involved in the process of replication, thus copying methylated strand from parent to newly synthesized strand. Ten-eleven translocation two family proteins can oxidize methylated DNA, the byproduct of which is 5-hydroxymethylcytosine. It is seen as promising in deepening the understanding of epigenetics and epigenetic-related diseases (36–38). Recently is proved that somatic mutations of DNMT3A and ten-eleven translocation two are associated with the appearance of clonal hematopoiesis. The latter is a well-known risk factor for the development of CVDs. Moreover, the same mutations were found in HF patients and further associated with rehospitalization and death (39, 40).

Histones are globular proteins subject to a plethora of modifications (41). Most of the covalent modifications that occur on histone are located on the flexible N-terminal portion (known differently as “tail”) that extrudes from the nucleosomal core and, depending on the specific mark involved, gene activity is also altered (42). Histone acetylation and phosphorylation are mostly transcription-activating; methylation and ubiquitination can either be transcription-activating or silencing, whereas sumoylation of specific amino acid residues acts as a transcriptional repressor. For instance, methylation of H3K9 or H3K27 generally marks silent chromatin, while methylation of H3K4 residues is associated with active chromatin (43, 44). The epigenetic composition of gene expression is well-regulated and involves the crosstalk between histone modifications and DNA methylation (45). Several chromatin-modifying enzymes are implicated in chromatin remodeling and PTMs histone modifications. Histone acetyltransferases (HATs or referred also as KATs) and histone deacetylases (HDACs) regulate the acetylation or deacetylation of lysine (Lys) residues. On the other hand, histone methyltransferases and lysine demethylases regulate methylation or demethylation of the same residues (46). Of note, histone modifications are associated with pathological processes eventually ensuing in disease states. Specific modifications can be linked to gene silencing or activation, as the example of mono-methylation of H3K4me activates the pro-inflammatory complex, nuclear factor kappa-B (NF-κB) (47, 48). More importantly, numerous studies have directly associated specific histone patterns, namely H3K27ac, H3K4me3, H3Kme1, H3K9ac, and H3K36me3 with HF development (49).

## Non-Coding RNAs

ncRNAs, unlike the other epigenetic regulators, are nucleic acid-based molecules that variously modulate gene expression. Regulation by small ncRNAs is another mechanism for epigenetic regulation of gene expression. Means by which ncRNAs govern gene expression can be post-transcriptional silencing, interference with transcriptional machinery, and regulation of splicing. ncRNAs are classified in small ncRNA, consisting of three main components: microRNAs (miRNAs), small interfering RNAs (siRNAs), piwi-interacting RNAs (piRNAs), and long ncRNAs (lncRNAs) (50).

### Small Non-coding RNAs

The small ncRNAs modulate gene expression through direct binding to coding or non-coding sequences of genes contributing to gene silencing, histone modification, DNA methylation targeting, and heterochromatin formation (51, 52). Compared to siRNAs and piRNAs, the role of miRNAs has been broadly studied in heart diseases. The miRNA pathway utilizes the RNA interference machinery (Dicer and Ago proteins). miRNAs typically bind to the 3′ UTR of target mRNAs and repress the mRNAs without cleaving RNAs thus affecting the protein levels of specific genes (51) miRNA-133 and miRNA-131 are inversely correlated to cardiac hypertrophy (53). miR-21 was found to be up-regulated during cardiac stress. It modulates genes like matrix metalloprotease-2 and transforms growth factor β1 receptor III, therefore playing an essential role in HF (54, 55). Extensive studies are needed to better define their role and to develop miRNA profiled that differentiate between HF patients with preserved ejection fraction and reduced ejection fraction (HFpEF vs. HFrEF).

### Long Non-coding RNAs

Numerous functional roles have been attributed to lncRNAs and further evidence has emphasized the role of lncRNAs in the mediation of epigenetic control over gene expression. Their regulatory affair is mediated by allosterically coupling binding domains with the switching of structural conformations and thereby activating or suppressing linked functional domains (56). Studies have highlighted the importance of these ncRNAs in heart development, physiology, and disease. Modulation of important cardiac transcription factors, such as Foxf1, Pitx, NKX2 and 5, and Irx3, are associated with Foxf1 adjacent non-coding developmental regulatory RNA (57). In a study that employed a comprehensive myocardial infarction-associated transcript, MIAT lncRNA was found overexpressed in left ventricular specimens from patients with end-stage HF (58). Moreover, lncRNA H19 was found dysregulated in hypertrophic hearts and its modulation prevented LV remodeling by disrupting the pro-hypertrophic NFAT signaling (59). The lncRNA NRON exhibited pro-hypertrophic effects in the murine hearts, and was identified as a cardiac hypertrophy promoter in a recent study (60). An interesting mechanism by which lncRNAs can induce gene activation is by reducing the activity of endogenous gene inhibitors such as miRNAs. For example, ncRNA-Mhrt prevented hypertrophy by orchestrating miR-145a-5p levels and subsequent expression of the KLF4/myocardin axis (61). Restoration of Mhrt expression protects the heart not only from cardiac hypertrophy but also from HF (62). Along the same line, lncRNA cardiac hypertrophy-related factor (Chrf) exerts anti-hypertrophic actions by modulating miRNA-489 levels and subsequent expression of the myeloid differentiation primary response gene (Myd88), a key player in LV hypertrophy (63).

## Existing Drugs With Cardio-Epigenetic Potentials

We know that epigenetics changes as we age and although our cells carry exactly the same genes, they look and act differently. The same can be said regarding the patterns of epigenetic modification that vary among individuals, tissues, and in different cells within the same tissue. Noteworthy, the advantage of a cell-specific drug in the context of CVD will be the narrowing of the broader effects drugs may have on other cell types in the same tissue/organ or other tissue or organs in the body. This limitation can be addressed through advances in techniques targeting specific cell types and gene loci. There is an emerging role of RNA therapies in this direction. For example, the synthetic siRNA Inclisiran exerts a liver specific suppression of PCSK9 expression and has a low side effect profile. In a randomized, single-blind, placebo-controlled, phase 1 trial was observed a significant reduction in circulating PCSK9 and LDL-C levels on hypercholesterolemia patients, thus leading to a reduction of cardiovascular risk (64). Although cell specific epigenetic therapies are still lacking in CVD patients, recent clinical trials have shown that systemic targeting of specific miRs improves cardiac damage in HF (65). Indeed, a first-in-human evidence phase 1b randomized, double-blind, placebo-controlled clinical study, recently showed that miR-132 inhibition was safe and led to leading to positive trends in myocardial fibrosis markers. The use of the first miRNA drug, CDR132L, was associated with promising beneficial effects in patients with chronic HF (66). Despite the cells in our bodies have identical genome, it is thought they can differentiate distinct tissues and organs *via* cell-specific variations in gene expression. This variation in gene expression can be further used to design specific drugs. Therefore, more than on cell-specific way, epi-drugs act upon the epigenetic changes that occur in a particular disease to a particular tissue, organ, or system. Still, it remains a challenge to a successful epigenetic drug design since epigenetic enzymes often work in multimeric complexes and this complicates the translation of *in vitro* efficiency to *in vivo* efficacy. Epi-drugs can have broader effects; therefore, this is considered as a limitation of epigenetic therapy. However, technical advances facilitating specific epigenetic editing may provide solution to address this limitation. Considering the galloping steps in the epigenetic and epigenetic pharmacology fields, we still can’t know how far we are as it is the limitation of the research and therapies with the ability to modulate the epigenome are still limited. Over the last few years, several widely used and very well-known drugs are endowed with epigenetic effects (Table 1). For instance, despite the broad usage of metformin as a first-line medication for the treatment of type 2 diabetes (T2D), it was fascinating to find out that this drug can also act as an epi-drug. It is already reported the beneficial role of metformin in lowering the risk of CVDs mortality and cancer, however, its role as an epigenetic editing drug was reported much later (67, 68). In a follow-up study including T2D and hypertensive patients, long-term prescription of metformin was effective in improving left ventricular diastolic function and hypertrophy, thus, decreasing the incidence of new-onset HFpEF (69). It is proposed that metformin promotes phosphorylation and activation of AMPK, thus inhibiting the gluconeogenic genes. AMPK is a major regulator of cellular metabolism, and its activation impacts numerous pathways, including epigenetic processes (70). Through AMPK activation and consequently histone modifications, this drug, promotes either increase or decrease in the expression of several genes Metformin also indirectly increases HAT1 activity. This was observed in a mouse embryonic fibroblast model where HAT1 phosphorylation occurred *via* AMPK (71). By contrast, metformin treatment led to reduced activity of two important co-activators of multiple genes involved in inflammation and glucogenesis, HATs p300 and the CREB-binding protein. Both phenomena are highly represented among HF patients (72–74). Another group of novel epi-drugs worth mentioning is sodium-glucose co-transporter-2 inhibitors (SGLT2I). It is reported not only their role in lowering the risk of HF hospitalization in T2D patients but also in lowering all-cause mortality, regardless of the presence of diabetes (75). The SGLT2i dapagliflozin was recently shown to exert cardiac and renal protective effects. The drug modulates important miRNAs involved in the pathophysiology of HF, such as miR199a-3p and miR30e-5p that are involved in the regulation of PPARδ levels mitochondrial fatty acid oxidation (76, 77). The SGLT2i empagliflozin was also reported to increase renal protection and prevent cardiac fibrosis, thus improving cardiac hemodynamics in experimental models of HF (78–81). One of the most studied mechanisms in the development of HF is DNA methylation. Hydralazine – a recent vasodilator with beneficial effects in HF – was also shown to exert epigenetic effects by decreasing the expression of DNA methyltransferase 1. Moreover, this drug can modulate calcium homeostasis in cardiomyocytes by decreasing promoter methylation of SERCA2a while enhancing SERCA2a protein and activity (82). Epigenetic effects of this drug are also linked with specific gene expression through methylation of CpG islands in the gene promoters (83). Statins are known to upregulate the levels of miRNA-221/222 (downregulation of which is related to coronary heart disease) (84), upregulate levels of miR-22 (involved in angiotensin II-mediated cardiac hypertrophy) (85), restore the levels of miRNA-483 (modulator of CTGF *via* Krüppel-like factor 4) (86), and modulate several others (87). They can reverse subtelomeric methylation of DNA in T2D patients (88) and provoke epigenetic changes through Sirt1 transcription modulation. The latter is linked with the regulation of inflammatory and apoptotic mechanisms, which are important in cardiovascular risk prevention (89).

**TABLE 1:** Widely used drugs with known epigenetic effects and potential use in HF.

Drug name	Pharmacological effect	Epigenetic action	Potential application for HF prevention and treatment
**Statins** (162–167)	- reduce cholesterol biosynthesis through inhibition of HMG-CoA reductase	- DNA methylation - histone acetylation - ncRNA expression	Regression of atherosclerosis through H3 and H4 acetylation; reduce mortality in acute HFpEF patients without coronary heart disease; prevent endothelial senescence *via* enhancing SIRT-1 expression; modulate eNOS expression in premature myocardial infarct patients; downregulate miR-146a/b in CAD patients; prevent ED by downregulating miR-221-5p, miR-27b-3p, and miR-16-2-3p
**Apresoline (Hydralazine)** (81, 168)	- lowers high blood pressure by relaxing resistance arterioles	- decrease the expression of DNA methyltransferase 1	Decreases vascular resistance; improves cardiac function; modulates calcium homeostasis in cardiomyocytes through DNA hypomethylation
**SGLT2 inhibitors** (169–173)	- reduce renal tubular glucose reabsorption	- DNA methylation - post transcriptional modification of histones -miRNA regulation	Promising for the prevention and treatment of diabetic cardiomyopathy; improve the NO-sGC-cGMP-cascade thus attenuating myocardial oxidative stress and cardiac inflammation; reduce cardiovascular mortality in HFrEF and HFpEF; exhibit cardiovascular benefits by increasing the circulating and tissue levels of β-hydroxybutyrate; improve hemodynamics in HF
**Metformin** (68, 71–73, 174, 175)	- improves glucose tolerance and increases peripheral glucose uptake and utilization	- post transcriptional modification of histones	Improves LV diastolic function; prevent HF through inhibition of p300 HAT activity; reduces mortality in HF patients; improves cardiac fibrosis by TGF-beta(1)-Smad3 inhibition; facilitates ED by enhancing SIRT1 and AMPK expression in endothelial cells
**Tranylcypromine** (115, 176, 177)	- helps in depressive episodes by irreversible inhibition of the enzyme monoamine oxidase	- LSD1 inhibitor	Vascular repair; improves angiogenesis; LSD1 inhibition prevents cardiomyopathy and improves heart functioning
**Trichostatin A** (178–181)	- antifungal and antibiotic	- reversible inhibitor of class I and II histone deacetylase enzymes	Prevents and reverses atrial fibrosis blunting connexin40 expression; modulates c-kit signaling thus preventing cardiac remodeling and dysfunction while promoting myocardial repair; attenuates cardiac hypertrophy by reducing H3K9/K14ac; suppresses pro-inflammatory NFκB target genes by broad histone deacetylation

Other compounds with epigenetic effects on HF are folates, omega-3 polyunsaturated fatty acids (PUFAs), and some organosulfur compounds. Folates are known for having a role in the prevention of CVD, although the exact mechanisms are yet to be elucidated (90, 91). They are differently known as methyl donors able to generate S-adenosylmethionine. This serves as a methyl-donor for methyltransferase needed for the methylation of cytosine in DNA or lysine in histones (92). Folate deficiency can lead to global DNA hypomethylation, which is strongly related to CVDs (93), or to endothelial dysfunction, as they act as epigenetic regulators of the transcription of the mitochondrial adaptor p66^Shc^ (94). P66^Shc^ is a crucial driver of myocardial dysfunction which is strongly related to HF (4, 95, 96). PUFAs on the other hand, are seen as effective in managing, preventing, and lowering the risk of death in HF patients (97, 98). For instance, a very recent study showed that lower plasma levels of docosahexaenoic acid had a significantly higher incidence of all-cause death in HFpEF patients (99). Moreover, PUFAs are associated with an impact on cardiac fibrosis, cardiac remodeling and function, and a lower risk for recurrent HF hospitalization (100–102). DNA methylation-sensitive mechanisms are held responsible for PUFAs’ effects on the epigenome (103). The organosulfur compounds are mostly represented by *Brassicaceae* botanical family and are recognized for their beneficial effects on the cardiovascular system (104–106). Evidence shows they modulate gene activity through posttranslational covalent modification of nucleosome histone proteins. For instance, the well-known representative of this group, sulforaphane, acts through direct downregulation of HDAC enzyme activity in different cell types (Table 2) (107). It was previously reported to modulate the Nrf2 pathway, thus preventing fibrosis and vascular remodeling (108). Through the inhibition of DNMTs and HDACs, sulforaphane reduced methylation levels while enhancing Ac-H3 enrichment on the Nrf2 promoter. Epigenetic remodeling of the Nrf2 promoter may lead to sustained upregulation of the transcription factor with subsequent antioxidant effects on the failing heart (109).

**TABLE 2:** Natural compounds with known epigenetic effects and potential use in HF.

Compound name	Epigenetic action	Potential application for HF prevention
**Resveratrol** (187, 188)	- Class I, II & IV HDAC inhibitor	Deacetylates NFkB-p65 and H3 thus attenuating cardiac oxidative stress, hypertrophy, and ED; beneficial effects in ischemic heart disease
**Curcumin** (189, 190)	- HAT inhibitor	Improves endothelial function; prevent HF through inhibition of p300 HAT activity; reduces atherogenic risk in T2D patients; prevents myocardial infarction by lowering inflammation and increasing SIRT1levels;
**Danshen** (191)	- HMT inhibitor	Attenuates cardiac hypertrophy by reducing H3K9 trimethylation and FHL1 up-regulation; beneficial effects in LV remodeling
**Sulforaphane** (107, 192–194)	- Class I, IIa & IIb HDAC inhibitors	Improves microvascular endothelial function; attenuates oxidative stress and inflammation through Nrf2 activation and TNF-α downregulation;
**Epigallocatechin gallate** (195–197)	- DNMT inhibitor - HAT inhibitor - miRNA regulator	Restores autophagy; attenuates doxorubicin-induces cardiotoxicity through anti-inflammatory and anti-apoptotic effects in heart; lowers oxidative stress levels; anti-fibrotic agent
**Caffeic acid** (198)	- Class I & II HDAC inhibitor - Class III HDAC expressor	Attenuates cardiac dysfunction and fibrosis, lowers mitochondrial oxidative stress through modulation of SIRT1 and SIRT3; prevents cardiac remodeling *via* down-regulation of the MEK/ERK modulation

It is known that sex can influence cardiovascular epigenetics thus highlighting a need of considering sex-based epi-drugs for the modulation of HF-related features. To date only few exploratory studies are available and additional investigations are required to better understand how sex (and gender) affect the epigenetic landscape in cardiac cells. Sex-differences in the epigenetic regulation of myocardial hypertrophy and inflammation would also imply a sex-based, personalized approaches when designing epi-drugs. For example, an interplay between sex hormones and sex-specific epigenetic mechanisms has shown to underline the gender dimorphism in HF prevalence. Estrogen and androgen receptors bind to hormone response elements and recruit the histone acetyltransferases CREB binding protein (CBP) and E1A binding protein p300 (EP300) to the DNA (110) strongly suggesting that sex hormone may directly regulate DNA and histone-modifying enzymes to impact the epigenetic processes in a sex-specific manner in the development of the spectrum of HFpEF and its course. Future studies will help to tailor epigenetic-editing interventions also based on sex and gender.

## Promising Novel Epi-Drugs With Potential Use in Heart Failure

Epigenome’s flexibility has led to the exploitation and development of a variety of epigenetic compounds (Table 3), many of which are already approved by the FDA for the treatment of different diseases. These drugs cause epigenetic changes by targeting at least one of the three main epigenetic mechanisms: DNA methylation, histone modification, and ncRNA. The first documentation on epi-drugs dates with the DNA methyltransferase inhibitors, 5-azacytidine, and 5-aza-2′-deoxycytidine. Both these compounds have a high structural similarity with cytidine. Additionally, structurally similar analogs were seen as antimetabolites capable of interfering with the normal function of the natural ones (111). Later, HDCA inhibitors like Romidepsin and hydroxamic acid were discovered and with them, a myriad of new epigenetic compounds is now being developed and tested, thanks to active research in the field (112). In the next paragraphs, we will focus on epi-drugs with an impact on CVDs, with emphasis on HF treatment.

**TABLE 3:** Epi-drugs with potential application in heart failure.

Drug name	Epigenetic action	Potential application for HF prevention and treatment
Apabetalone (RVX-208) (141–143)	- BET inhibitor	- inhibits expression of pro-inflammatory cytokines (IL-6 and TNF-α); attenuates endothelial inflammation; impacts on microvascular dysfunction; improves angiogenesis; attenuates cardiomyocyte hypertrophy
JQ1 (144, 175)	- BET inhibitor	- prevents hypertrophy and profibrotic myocardial signaling by inhibiting NF-κB and TGF-β signaling; impacts positively on LV remodeling and diastolic dysfunction; prevents HF hallmarks like LV fibrosis, and cardiomyocyte hypertrophy
Zolinza^®^ (Vorinostat) (176, 182)	- HDAC inhibitor	- attenuates cardiovascular remodeling; reduces inflammatory cytokine levels by modulating gene expression related to inflammatory response; blunts myocardial hypertrophy; preserves cardiac function in animal models of HF; improves cardiovascular structure and function; improves cardiac function after MI by inhibiting HDAC6 activity
Givinostat (183, 184)	- HDAC inhibitor	- improves post-AMI cardiac dysfunction; reduces cardiac fibrosis; prevents cardiac remodeling
Apicidin (181)	- HDAC inhibitor	- reduces LV hypertrophy and failure; induces LV relaxation; prevents cardiac hypertrophy
Remlarsen (MRG-201) (146, 185)	- miR-29 mimic	- reduces cardiac fibrosis and collagen expression; improves LV relaxation
RG-012 (186)	- miR-21 antagomir	- beneficial effects on LV relaxation; prevents cardiac inflammation; attenuates LV remodeling after MI

### DNA Methyltransferase Inhibitors (DNMTi)

DNMT mediated DNA-methylation is a relevant mechanism underpinning CVDs, and it is one of the most studied epigenetic processes in the development of HF. Depletion of the methyltransferase DNMT3A in human cardiomyocytes affects their morphology, function, and metabolism (113) and leads to hypertrophy (114). Over the last years, we became familiar with two mains representatives of this class: azacytidine and decitabine. Both were approved by FDA under the names of Vidaza and Dacogen, respectively (115). Due to their ability to inhibit DNMT, they can be used as chemical tools to induce genome hypomethylation. A relevant example is the hypomethylation of genes implicated in vascular homeostasis, such as endothelial nitric oxide synthase. Treatment of endothelial cells with DNMT inhibitors was found to restore nitric oxide synthesis and bioavailability with potential beneficial effects on micro-and macrovascular endothelial function *in vivo* (116). Rescuing microvascular dysfunction with epi-drugs could be relevant in the setting of HF given the pivotal role of detective endothelial function in its pathogenesis, especially in HFpEF (117). Along the same line, the vasodilator hydralazine was recently found to act *via* an epigenetic mechanism (DNMT1 inhibition). This drug can modulate calcium homeostasis in cardiomyocytes by decreasing promoter methylation of SERCA2a while enhancing SERCA2a gene expression and activity (82).

### Histone Deacetylase Inhibitors (HDACi) and Histone Methyltransferase Inhibitors (HMTi)

Based on their structure, HDACs are categorized into four major classes (I-IV) (118). The regional modifications of chromatin by HDACs may activate or silence specific genes and these enzymes are found dysregulated in numerous diseases, including CVDs (119). Early insights on the potential of this class of drugs in the cardiovascular setting come from experimental studies in mice, where HDAC inhibition was able to prevent pressure overload-induced cardiac hypertrophy (120). Moreover, in experimental models of myocardial ischemia-reperfusion injury, HDACi were shown to reduce infarct size by 50%, prevent pathologic remodeling of the LV to limit the extent of cell death at reperfusion while restoring autophagic flux (121, 122). Other studies have associated this class with the modulation of genes involved in cardiac fibrosis, hypertrophy, mitochondrial biogenesis, and inflammation, key features of the failing heart (123–125). Several HDACi have shown beneficial effects on the CV system. The first approved drugs with an influence on epigenetic post-translational modification of histones are vorinostat and romidepsin (112). Vorinostat is a hydroxamic acid HDACi firstly used in cancer therapy which only later started gaining attention in the cardiovascular field (126). Romidepsin is a more specific class I HDACi, while most compounds of this class are considered pan-HDACis since they inhibit class I, II, and IV HDACs (127). HFpEF is characterized by LV diastolic dysfunction and increased filling pressures. The HDACi givinostat improved cardiac performance in two different experimental models of diastolic dysfunction (128). Among the first compounds reported as HDACi is butyric acid. This compound gained more attention when two already approved drugs, namely valproic acid and phenyl butyric acid used for the treatment of epilepsy and urea cycle disorders, respectively, were identified as HDACi (129–131). Butyric acid was shown to inhibit cardiac expression of pro-hypertrophic and pro-inflammatory genes, whereas valproic acid protected against myocardial infarction-induced LV remodeling *via* epigenetic modulation of the Foxm1 pathway (132, 133).

HMTs are the enzymes that post-translationally add one to three methyl groups to lysine residues in proteins. To date, we know few inhibitors which entered clinical trials for the treatment of several clinical conditions, namely the DOT1L inhibitor for the treatment of leukemia, tazemetostat for B cell lymphoma, or EPZ015938 for cancer treatment (134, 135). Among different HMTi, only tanshinone IIA (one of the main active principles of Danshen) has shown a direct involvement in CVD. This nutraceutical compound was shown to reduce H3K9 trimethylation *via* inhibition of the methyltransferase JMJD2A. These chromatin changes were associated with epigenetic silencing of pro-hypertrophic genes and prevention of maladaptive cardiac remodeling in experimental studies. Additionally, tanshinone IIA elevated Nrf2 expression by inducing Nrf2 promoter hypomethylation and inhibition of HDAC activity (136).

### BET-Inhibitors (BETi)

Bromodomains represent the first class of ∼110 amino acids reader proteins with a potential role in transcriptional regulation. The BET protein family (including BRD2, BRD3, BRD4, and BRDT) includes epigenetic reader proteins that bind specific acetylated lysine residues on histone tails where they facilitate the assembly of transcription complexes. BET inhibition, including the use of specific chemical BET inhibitors like JQ-1, has many reported effects *in vivo* in the cardiovascular setting, like preventing intimal hyperplasia, pulmonary arterial hypertension, and cardiac hypertrophy. Studies conducted over the last 5 years show that these reader proteins are implicated in multiple biological pathways, and studies appearing in rapid succession contribute to unveiling their role in the epi-drug discovery tableau (14). At the clinical level, the potential anti-inflammatory action of these drugs has been widely investigated in the context of clinical oncology (137–139). Recent evidence suggests that BETi could be beneficial in patients with CVD. Apabetalone (APA, also known as RVX-208) – an FDA-approved small molecule targeting BET proteins (namely BRD4) – was recently shown to interfere with several hallmarks of the atherosclerotic process such as lipid metabolism, oxidative stress, and vascular inflammation. Treatment of patients with dyslipidemia with APA led to up 6.7% increase in apolipoprotein A-I levels, a 6.5% increase in high-density lipoprotein cholesterol (HDL-C), and a 21.1% decrease in high-sensitivity C-reactive protein. The improvement in lipid profile was associated with fewer cardiovascular events among patients treated with APA than with placebo (140). In phase II trials APA reduced the relative risk of major adverse cardiac events (MACE) in patients with established CVD by 44% and in diabetic patients by 57%. The recent phase III BETonMACE trial, designed to investigate the impact of APA on cardiovascular outcomes in 2,425 patients with diabetes after an acute coronary syndrome, failed to meet the primary endpoint (cardiovascular death, non-fatal myocardial infarction, or stroke), but showed a favorable trend (18% difference in relative risk), indicating that APA might be a promising treatment option and suggesting that larger clinical trials are needed in this patient population. Of interest, APA was associated with a reduced risk of first (29 vs. 48, *P* = 0.03) or first and recurrent congestive HF hospitalizations (35 vs. 70) (141). Undoubtedly, larger clinical trials are needed to explore better the safety and efficacy of apabetalone for the treatment of CVD and its potential use in the setting of HFrEF and HFpEF (142–144). Together with APA the BETi JQ1 was shown to prevent pressure-overload induced hypertrophy and HF. In this study, t JQ1 treatment blocked the transactivation of a subset of stress-inducible genes, particularly involved in the transforming growth factor-β (TGFβ) and nuclear factor-κB (NF-κB). Both pathways are important for pathological LV remodeling and the transition to HF (145).

### ncRNA Therapeutics

Therapeutic approaches targeting ncRNAs (by mimics or antagomiRs) represent a new frontier in HF treatment. Evidence has reported the beneficial role of miR-21 antagomir and miR-29 mimic, named, respectively, RG-012 and MRG-201. The latter one is a synthetic microRNA mimic approved by the FDA and known as Remlarsen. According to the first clinical data reported from preclinical studies, Remlarsen was endowed with an anti-fibrotic effect in the cornea (146). A very recent study showed the anti-fibrotic effect of this mir-mimic in lung fibroblasts and precision-cut lung slices. Remlarsen resulted effective by decreasing COL1A1 and ACTA2 gene expression and reducing collagen production. Also, its anti-fibrotic activity is being tested in phase I clinical trial (147). This synthetic compound can be of interest in complications associated with fibrosis such as myocardial fibrosis in patients with HF (148). On the other hand, miR-92 mimics may help in modifying transcriptional networks regulating angiogenic responses, ECM remodeling, cardiac fibrosis, hypertrophy, and myocyte growth. Additionally, MRG-110, an antagonist of miR-92a, is currently being investigated in phase II clinical trials in patients with ischemic cardiomyopathy and HF (149). Last year, a Phase 1b study (NCT04045405) reported fascinating data on a new antagomir of miR-132. It is known that miR-132 activation leads to adverse remodeling and pathological hypertrophy, whereas inhibition of this miRNA by CDR132L, a synthetic oligonucleotide inhibitor, showed to be effective and safe in patients with ischemic HF (150).

Modulation of lncRNAs is also a promising strategy to prevent cardiac remodeling and HF. Mounting evidence indicates a prominent role of lncRNAs in cardiovascular dysfunction. For instance, lncRNAs like MALAT1, STEEL, MANTIS, and MEG3 regulate endothelial functioning being involved in cell proliferation, migration, apoptosis, and angiogenesis (151–154). Others like SMILR, MYOSLID, and SENCR regulate vascular smooth muscle cell phenotypes, proliferation, migration, and apoptosis (155, 156). Many others serve as biomarkers in many CV conditions such are atherosclerosis, coronary artery disease, LV dysfunction, cardiac hypertrophy, and myocardial dysfunction (157).

An emerging reversible post-translational modification occurring in various RNA molecules, such are mRNA, miRNA, lncRNA, tRNA, etc., is RNA methylation. This type of modification is identified to have contributed to the functional characterization of different CVDs. The m^6^A RNA methylation is not only important for normal heart function but it is strongly related to HF development. Alterations of gene expression during HF are accompanied with increased levels of m^6^A RNA methylation (158, 159). Of interest, a recent study showed that the expression of m^6^A writers METTL3, METTL4, and KIAA1429; m^6^A eraser FTO; and reader YTHDF2 was up regulated in HFpEF patients, compared with health controls. Furthermore, the expression of FTO was also elevated in HFpEF mice (160). Gene Ontology analysis revealed that protein folding, ubiquitin dependent ERAD pathway, and positive regulation of RNA polymerase II were the three most significantly altered biological processes in HFpEF. These new findings suggest that the modulation of epi-transcriptomic processes, such as m^6^A methylation, might be an interesting target for therapeutic interventions in HFpEF patients and could set the stage for personalized approaches in this setting.

The relationship between lncRNAs and CVDs needs further study. The discovery of techniques to manipulate their activity can help in improving therapeutic strategies in cardiac dysfunction and ameliorate HF pathological progression. Besides their role in CVD, lncRNAs may also act as causal biomarkers and may help distinguish among different conditions, thus helping in differential diagnosis. The combination of classic biomarkers with lncRNA signatures is indeed emerging as a novel and promising tool to stratify the risk of developing CVD and HF (161).

## Conclusion

Even though epigenetic modifications acquired during the lifetime can be passed to the progeny, still these changes are reversible and amenable to pharmacological intervention. Recent evidence suggests that targeting epigenetic changes is possible and relatively safe. Indeed, clinical studies conducted so far in cancer patients did not show significant effect of these drugs on the incidence of second malignancies or any other severe, unanticipated toxicity. That having said, most available epi-drugs lack specificity toward selected transcriptional programs and cell types. Cell-specific epigenetic editing by epi-drugs is the next challenge in pharmaceutical research. The recent unveiling of new epigenetic compounds is of paramount importance given the paucity of available treatments for HF patients, especially those with HFpEF. Further research is needed to better define selective epigenetic therapies able to selectively modulate transcriptional programs in specific cell types (i.e., cardiomyocytes, fibroblast, endothelial cells). A better definition of these important aspects will shed light on the importance and potential effectiveness of epi-drugs in the setting of HF.

## Author Contributions

FP, SC, and EG conceptualized the manuscript. EG wrote the manuscript. FP guided the writing process. SM assisted in drafting the manuscript. SA assisted in organizing and shaping the figure. VC, SC, and FP revised the manuscript critically and provided important intellectual content. All authors have contributed significantly.

## Conflict of Interest

The authors declare that the research was conducted in the absence of any commercial or financial relationships that could be construed as a potential conflict of interest.

## Publisher’s Note

All claims expressed in this article are solely those of the authors and do not necessarily represent those of their affiliated organizations, or those of the publisher, the editors and the reviewers. Any product that may be evaluated in this article, or claim that may be made by its manufacturer, is not guaranteed or endorsed by the publisher.
